# Interventions aiming to improve menstrual, sexual, reproductive, and mental health among out-of-school girls: a systematic review

**DOI:** 10.3389/fpubh.2024.1440930

**Published:** 2024-12-04

**Authors:** Karinn Farquharson, Alexandra Quinn-Savory, Garazi Zulaika, Linda Mason, Susan Nungo, Elizabeth Nyothach, Holger Unger, Muthusamy Sivakami, Philip Spinhoven, Penelope A. Phillips-Howard, Anna Maria van Eijk

**Affiliations:** ^1^Department of Clinical Sciences, Liverpool School of Tropical Medicine, Liverpool, United Kingdom; ^2^Center for Global Health Research, Kenya Medical Research Institute, Kisumu, Kenya; ^3^Global and Tropical Health Division, Menzies School of Health Research, Charles Darwin University, Darwin, NT, Australia; ^4^Department of Obstetrics and Gynaecology, Royal Darwin Hospital, Tiwi, NT, Australia; ^5^School of Population and Global Health, Tata Institute of Social Sciences, Mumbai, India; ^6^Department of Psychiatry, Leiden University Medical Center, Leiden, Netherlands

**Keywords:** out-of-school girls, sexual and reproductive health, mental health, menstrual health, knowledge

## Abstract

**Purpose:**

Out-of-school adolescent girls (OoSGs) can lack education on menstrual, sexual, reproductive, and mental health (SRMH) and be more vulnerable to SRMH harms. Targeted interventions could reduce these risks. We assessed interventions and their effectiveness among OoSGs globally.

**Methods:**

Six online databases were searched for interventional studies targeting SRMH problems in OoSGs. Two coders independently abstracted data from each eligible study, following the Preferred Reporting Items for Systematic Reviews and Meta-Analyses guidelines. We summarized results using forest plots.

**Results:**

A total of 1,244 studies were screened; eight studies with sufficient information on SRMH outcomes were included (9,084 OoSGs, range 100–3,026 per study, overall study quality low-to-moderate). Six were educational programmes, and two were cash interventions; no study was directed at mental health. Participants were recruited through village census, outreach workers, and work locations. Some improvements were seen in indicators of SRMH knowledge (four studies), attitudes (one study), and risky sexual behaviors (four studies); however, no reduction was seen in human immunodeficiency virus or herpes simplex virus-2 incidence (two studies).

**Discussion:**

This review suggests that programmes can improve OosG’s SRMH knowledge, attitudes, and practices and can be delivered in low resource contexts. Effective interventions are needed to support OoSGs, including interventions focusing on SRMH support incorporating elements of cash-transfer, and comprehensive sexual education, and to aid local policy and programming.

## 1 Introduction

Adolescence spans the ages of 10–19 years and globally, approximately 600 million girls fall within this age group, of whom 89% live in low-and middle-income countries (1). Adolescence is a critical time of psychological and biological change, with girls adapting to pubertal changes and navigating menstruation and the potential risks associated with sexual exposure [e.g., gender-based violence, sexually transmitted infections (STIs), and pregnancy]. While improvements in education have been reported globally, in many low-and middle-income country settings the prevalence of girls not in education or training remains high, leaving them with few opportunities to enter the workforce (2). In many low-and middle-income countries, girls are unable to attend or remain in school because of poverty, child marriage, or unintended pregnancies (3–6). Worldwide, an estimated 244 million children and adolescents between 6 and 17 years of age were out of school in 2021, with female out-of-school rate 4.2 percentage points higher than the male rate in sub-Saharan Africa (7).

School enrolment has been found to be strongly associated with improved sexual, reproductive, and mental health (SRMH) outcomes and healthcare utilization (8). Studies have found that out-of-school girls experience earlier sexual debut, more frequent sex with multiple partners, less condom and contraceptive use, and higher prevalence of STIs (including HIV) (9, 10). Moreover, out-of-school girls marry earlier and enter motherhood on average 2.5 years earlier than girls with secondary education or higher (9). Further, being out-of-school has been identified as a potential risk factor for mental health problems and suicidal ideation among girls in low-and middle-income countries (11); and such girls are less likely to access healthcare and to be reached by health campaigns relative to their school-going peers (12).

A lack of comprehensive sexuality education (CSE), encompassing physical, cognitive, emotional and social aspects of sexuality, including puberty development and sexual health can lead to unprotected sexual exposure, resulting in STI, including HIV, and pregnancy; in one study, girls without education were found to be at double the risk of HIV compared with girls with some level of education (13, 14). Education is recognized as an essential determinant of health and productivity within the United Nations’ Sustainable Development Goal (SDG, goal 4). Additionally, SDG 3 (ensure healthy lives and promote wellbeing) includes target 3.3, dedicated to combatting HIV/AIDS. To achieve target 3.3, including the promotion of safe SRH behaviors and resultant outcomes, out-of-school girls must be included in high quality, inclusive and transformative education about condom use and HIV/AIDS (15).

Most adolescent health promotion programmes are initiated and monitored in schools (16). However, these ignore the needs of adolescents who are out-of-school and are at highest risk of SMRH harms. Low educational attainment among out-of-school girls is reported to have an effect on girls’ immediate health and an extended effect on their future health and the health of their children (17). The high vulnerability of out-of-school girls require intervention through supportive health programmes and targeted solutions (18); however, it is unclear what interventions have been tested among out-of-school girls, and how effective they have been (19).

We conducted a systematic review of interventions targeting out-of-school girls’ menstrual, sexual, reproductive, and mental health and their effectiveness. This review also explored the approaches used to deliver these interventions outside of the school settings.

## 2 Methods

This review was registered in Prospero (CRD42022274402).

### 2.1 Eligibility

Studies were included if they presented results on an intervention to improve menstrual, sexual reproductive or mental health among out-of-school girls. Quantitative studies with a before/after design, and randomized, non-randomized or quasi-randomized trials were eligible. Purely qualitative studies were ineligible. Studies whose interventions also targeted community health workers, teachers, boys and girls, or among in-and out-of-school study populations, were only included if the results were stratified for out-of-school girls. We used the definition of out-of-school girls as defined within the identified articles. Many studies included young women aged 18–24 years; these studies were not *a-priori* excluded.

### 2.2 Information sources

Sources for the search for relevant literature included CINAHL, Global Health, Medline, PubMed, Embase, Web of Science and APA PsycINFO. Additionally, reference lists of eligible material and review articles, trial registers, and conference proceedings were searched. No time or language restriction were incorporated in the search, and databases were searched from their inception. The last search was conducted on March 20, 2024. The search terms and strategies are available in the Supplementary Tables S1, S2.

### 2.3 Selection processes

All citations were imported into Excel and duplicates were removed. Two reviewers (AMvE and KF, or AMvE and AQ) independently screened the abstracts and full texts where available and agreed on final study eligibility. Disagreements were resolved by a third reviewer who served as the tiebreaker. A log of all studies excluded and reasons for exclusion was kept. Data from reports from studies with multiple publications were combined in one record, to avoid duplication of individual data.

### 2.4 Data collection processes

Two people (AMvE and KF, or AMvE and AQ) independently extracted data from the study and conducted a risk of bias assessment using the data collection form of the Cochrane collaboration (20). This form collects data on the study and a risk of bias assessment using seven categories, visualized using a risk-of-bias plot tool (21). Studies could obtain two points per category (maximum 14 points), and a score of 80% or above was considered good quality (for further information, see Supplementary Figure S1). Data items extracted included first author, journal, year of publication, study funding, conflict of interest, type of study, type of participants, type of intervention, and outcome measures. Outcomes included factors related to menstruation (use of menstrual product, disposal, knowledge about menstruation), sexual and reproductive health (knowledge of STIs and HIV, sexual behavior such as type of partners, use of condoms and contraceptives, pregnancy) and mental health measures (e.g., depression scales). Risk ratios (RR) and odds ratios (OR, or aOR for adjusted odds ratios) were used as effect measures for binary outcomes, and differences in means would have been used for continuous outcomes.

### 2.5 Synthesis

Study characteristics and method of recruitment were tabulated. Outcomes were categorized as attitude (e.g., would you visit a youth clinic), knowledge (e.g., do you know a youth clinic), behavior (e.g., have you visited a youth clinic) or emotions related (e.g., anxiety, depression, or post-traumatic stress). Interventions were categorized into main groups by type of intervention. Results were described in tables and presented in forest plots, without pooling of information into summary estimates, due to the wide variety in the type of interventions and outcomes reported. The numerators and denominators and measure of effectiveness (risk ratio, odds ratio, or adjusted estimates) were added to the plots (where available) to allow interpretation of the result. Where available, preference was given to adjusted estimates. The lack of comparability of outcomes prohibited any further analyses. Qualitative components of included studies, where available, were summarized.

## 3 Results

### 3.1 Characteristics

**Figure 1:**
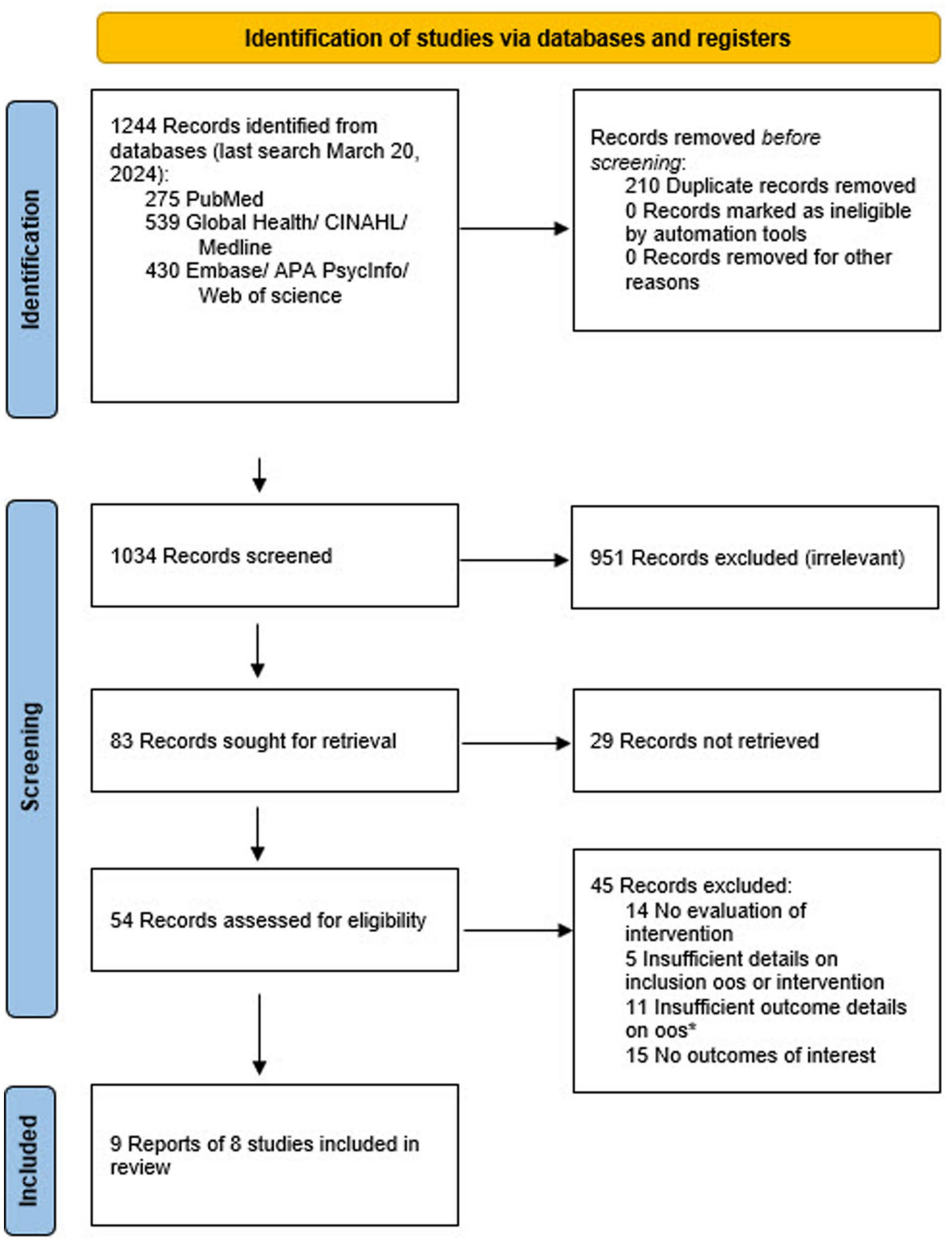
Prisma flow diagram showing study selection process. Oos: out-of-school girls.*Details of these studies are available in the Supplementary Tables S3–S5.

The database searches identified 1,034 records with potential information; after screening, 83 records remained. From these, 8 eligible studies including 9,084 participants were identified (Figure 1). Information on 11 excluded studies which reported inclusion of out-of-school girls but had insufficient information can be found in the Supplementary Tables S4–S6. The included studies were conducted in eight different low-and middle-income countries (China, Egypt, India, Democratic Republic of the Congo, Malawi, Nigeria, South Africa, and Tanzania) and varied widely in recruitment methods, interventions tested, and outcomes examined (Tables 1, 2; Supplementary Table S3). Four examined effects among out-of-school girls exclusively (22–25), two included both in-and out-of-school girls (26, 27), and two included males and females in-and out-of-schools (28, 29). Three were cluster-randomized trials; for two trials, adjusted estimates were included (25, 26). In the remaining cluster randomized trial, the sample size was small (50 participants in each arm), and only unadjusted results were presented (24). The overall study quality was moderate-to-low with only one study judged to be of good quality (Supplementary Figure S1).

**Table 1:** Characteristics of study included to evaluate interventions directed at sexual, reproductive, and menstrual health among out-of-school girls.

Author	Study design	Setting	Study period	Population for this review	Age, years, mean (SD)	Intervention^*^	Intervention duration	Timepoint of evaluation	Comparison	Sample intervention	Sample comparator	Outcome domains
Baird et al. (2010, 2012) (26, 39)	Cluster Randomized Trial	Zomba District, Malawi	2007–2009	Out-of-school girls, aged 13–22 years†	Intervention: 17.6 (2.2) Control: 16.8 (2.4)	Conditional cash programme	18 months	Behavioral: 12 months Serology: 18 months	Control group same population control clusters	223	226	HIV, HSV-2, syphilis prevalence Marital status Pregnancy Sexual activity, risky sexual behavior
Carney et al. (2019) (23)	Cluster Randomized Trial	Township, Cape Town, South Africa	2015–2016	Female school dropouts, aged 16–21 years	19.1 (1.6)	Group workshops	2 workshops 1 week apart	1 month after intake interview	Control group same population control clusters	50	50	Risky sexual behavior
Feng et al. (2020) (23)	Quasi-experimental	6 counties in China	2008–2011	Out-of-school girls, aged 12–24 years†	20.8 (NR)	Multisectoral cooperation mechanism	24 months	24 months	Control group from control counties	421	199	-Knowledge on presence and content of youth friendly service clinics
Kuringe et al. (2022) (24)	Cluster Randomized Trial	Shinyanga region, Tanzania	2017–2019	Out-of-school girls, aged 15–23 years	Median 20 (IQR 18–22)	HIV prevention and quarterly cash transfer	18 months	6, 12 and 18 months	Control group HIV prevention only	1,482	1,544	Incidence of HSV-2 infection HIV prevalence at follow-up Sexual activity and risky sexual behavior
Odeyemi et al. (2014) (22)	Quasi-experimental	2 markets in Lagos, Nigeria	Not reported, before 2014	Out-of-school girls, aged 10–19 years	17.1 (2.1)	Health education programme	5 education sessions	6 months post intervention	Control group from different market	332	350	Sexual health knowledge, behavior, and practice
Sieverding et al. (2016) (21)	Quasi-experimental	3 governorates in Northern Egypt	2009–2011	Out-of-school girls, aged 11–15 years	72.8% 12–14 years	Educational programme, reproductive health information	20 months	20 months	Control group same villages	1973	929	Reproductive health attitudes
Vayeda et al. (2021) (26)	Pre-and post-intervention	Narmada District, Gujarat, India	2018–2019	Out-of-school girls, aged 10–19 years†	16% 10–14 years 84% 15–19 years	Multi-level capacity building and education	7 awareness sessions over 1 year	12 months	Survey from same villages before intervention	243	236	Menstrual health knowledge and practice
Gayles et al. (2023) (28)	Quasi-experimental	2 communes in Kinshasa, Democratic Republic of Congo	2017–2018	Out-of-school girls, aged 10–14 years†	63% 10–12 years 37% 12–14 years	Multi-level programme utilizing a gender transformative approach	26 (1 h) sessions over 9 months	12 months post baseline (3 months post intervention end)	Control group random sample OOS adolescents from household listing	446	380	-SRH knowledge and communication

HIV, Human Immunodeficiency virus; HSV-2, herpes simplex complex virus 2; IQR, interquartile range; NR, not reported; SD, standard deviation; SRH, sexual and reproductive health.

^*^For a more detailed description of the intervention, see Table 2.

^†^Baird et al. (26) included in-and out-of-school schoolgirls. Feng et al. (28) included in-school and out-of-school boys/men and girls/women. Vayeda et al. (27) included both in-and out-of-school girls. Gayles et al. (29) included in-school and out-of-school very young adolescent boys and girls.

**Table 2:** Description of intervention.

Study	Country	Description of intervention
Baird et al. (2010, 2012) (26, 39)	Malawi	Conditional cash programme: cash transfers made monthly for a total of ten transfers per year split between guardian and girl. Household amount varied randomly (by use of computer-generated random numbers) by enumeration area, with monthly values of US$4, $6, $8, or $10. The amount received by the girl varied randomly between individuals, with monthly values of $1, $2, $3, $4 or $5, decided by drawing numbers from an envelope. For out-of-school girls (at baseline), the treatment group consisted of the conditional group only, where each payment was received if the girl attended school for 80% of the days that the school was in session during the previous month.
Carney et al. (2019) (23)	South Africa	Two group workshops of 2 hours each, delivered 1 week apart by trained female interventionists. Workshop one provided participants with information about HIV, AIDS, STIs, pregnancy and sexual risk behaviors. This workshop also taught sexual negotiation skills and correct condom use. Workshop two covered AOD use, gender power and violence, and education. Participants were also taught communication skills, specifically around conflict resolution. Formative FGD participant voices from targeted communities were incorporated into the body of the intervention and more practical activities around types of AODs and sexual risk activity, in addition to more information on teenage pregnancy, breastfeeding and reproductive health were added, as suggested by adolescents.
Feng et al. (2020) (27)	China	Multisectoral cooperation mechanism utilizing social network theory. Activities were based on stakeholder participation [multi-sectoral coordination meetings each year consisting of one nation level intensive training, >4 h curriculum on young people reproductive health (YRH) and youth friendly services (YFS), two lectures and Q&A activities, regular publicity, regular feedback from young people on services, one nation-level field technical supports and local self-assessments]; confidence (regular coordination meetings to discuss and resolve problems on YRH and YFS), improvements on YFS facilities, environment and quality to absorb young people, YRH education, trainings in multi-sectors including service providers, development and dissemination of various forms of IEC materials; and commitment/responsibility (development and issuing of local administrative documents on MSCM to clarify the role of each sector and cooperative activities, incorporation of each sectors performance in the cooperation into Annual review, coordination of local government to ensure workforce, funding and materials required, and technical support and assessment to identify and resolve problems timelines).
Kuringe et al. (2022) (24)	Tanzania	Core package of combination HIV prevention interventions, including risk reduction counseling, HIV testing services, condom use skills and provision, family planning counseling and service provision, sexually transmitted infection screening and treatment, gender-based violence interventions (escorted referrals and the desk for social, legal, and medical services provided to survivors of gender-based violence), tuberculosis and alcohol and drug abuse screening, and referral to services. Other features included SBCC training sessions and economic empowerment community banking (WORTH+) intervention. Participants in the intervention arm additionally received unconditional cash transfer in quarterly installments of 70,000 Tanzania shillings (~US$31) for 18 months through mobile money on a project-provided cellular phone while participants in the control arm additionally received cellular phones only.
Odeyemi et al. (2014) (22)	Nigeria	Education-entertainment programme in community center within the market. Peers attended five education-entertainment sessions with screening of films (that educated on risks and consequences of teenage pregnancy, promote abstinence, teach life skills, and provide information on contraception), health talks and interactive group sessions, drama sketch, training of peer counselors (30 adolescents over 1 week) and distribution of educational materials (such as pamphlets).
Sieverding et al. (2016) (21)	Egypt	Attendance of classes (about 30 girls) at local youth centre 3 hours per day, four times per week for 20 months; topics included literacy, basic math, sports, life skills such as gender roles, marriage, reproductive health, nutrition, hygiene, and rights. To foster a more supportive community environment for adolescent girls, a number of other programme elements, such as the formation of a village committee to inform community members about the broader issues of girls’ education and gender equity, community mobilization activities, and public events were included.
Vayeda et al. (2021) (26)	India	Multi-level capacity building and education. This consisted of capacity building of government frontline health workers and teachers, followed by supportive supervision. Meetings with stakeholders. “MHM-corners” and “MHM-Committees” were created at schools and Anganwadi-centres to improve access to menstrual absorbents and information. Seven awareness sessions for out-of-school girls by Anganwadi Worker. Improved access to absorbents and disposal facilities. It is possible that there is an overlap in participants between survey at enrolment and at endline, because participants were from the same villages.
Gayles et al. (2023) (28)	Democratic Republic of Congo	Multi-level programme utilizing a gender-transformative approach. This involved engaging very young adolescents, their families and communities in critical reflection of gender norms while recognizing social and institutional factors that influence very young adolescents’ gender attitudes, behaviors and norms. School and community-based clubs participated in 26 mixed-sex group sessions, held weekly for 60–90 min, over 9 months of the school year. Clubs used a toolkit consisting of interactive, age-appropriate materials. Supplemental activities to engage family, school, community and health system actors consisted of 6 hour-long reflective discussions, held monthly. To ensure alignment with government priorities and the local context, the intervention was implemented in partnership with community-based organizations and guided by a regional Ministry of Education and Health technical advisory group.

AID, acquired immune deficiency syndrome; AOD, alcohol and other drug; FGD, focus group discussions; HIV, Human Immunodeficiency virus; IEC, information, education and communication; MHM, menstrual hygiene management; MSCM, multisectoral cooperation mechanism; SBCC, social and behavior change communication; STI, sexually transmitted infection; YFS, youth friendly services; YRH, young people reproductive health.

### 3.2 Recruitment of out-of-school girls

Of the eight included studies, four used household surveys or listings to identify and recruit out-of-school girls, one study surveyed sub-sections within street markets in Nigeria to identify adolescent girls, one used a female outreach worker to recruit girls, another visited communities or worksites to recruit unmarried women, and one study used word of mouth, public announcements, and information through youth centers (Supplementary Table S3). The remaining study we classified as using a convenience sample in communities and workplaces without random sampling, as the authors stated “While, for out-of-school young people, the communities and enterprises that were willing to attend the survey and for which it was easy to organize young people were selected to recruit as many young people as possible” (28).

### 3.3 Type of interventions

Short descriptions of the included interventions are described in Table 2. Two were cash transfer programmes (monthly $1–5 for the girl on one and 4-monthly $31 in the other) and the others were SRH educational interventions (e.g., two-hour workshops on SRH, education sessions with films, classes at local health centers). Three education programmes had interventions not solely directed to the adolescent participants, with elements provided also for the participants’ community or service providers (27–29). For example, the study in India approached out-of-school girls, but also conducted capacity building among community health workers and teachers (27). One study in China had programmes for all levels of stakeholders, including youth friendly services, the health sector, and the local government (28). A study in the Democratic Republic of the Congo involved the community, with a programme of six one-hour monthly meetings. The duration of study interventions lasted from 1 month to 2 years, and evaluations were conducted after ending the programme or within 1–18 months post-intervention. No interventions to improve the mental health of out-of-school girls were identified.

### 3.4 Effect of interventions on sexual and reproductive health knowledge and attitude

**Figure 2:**
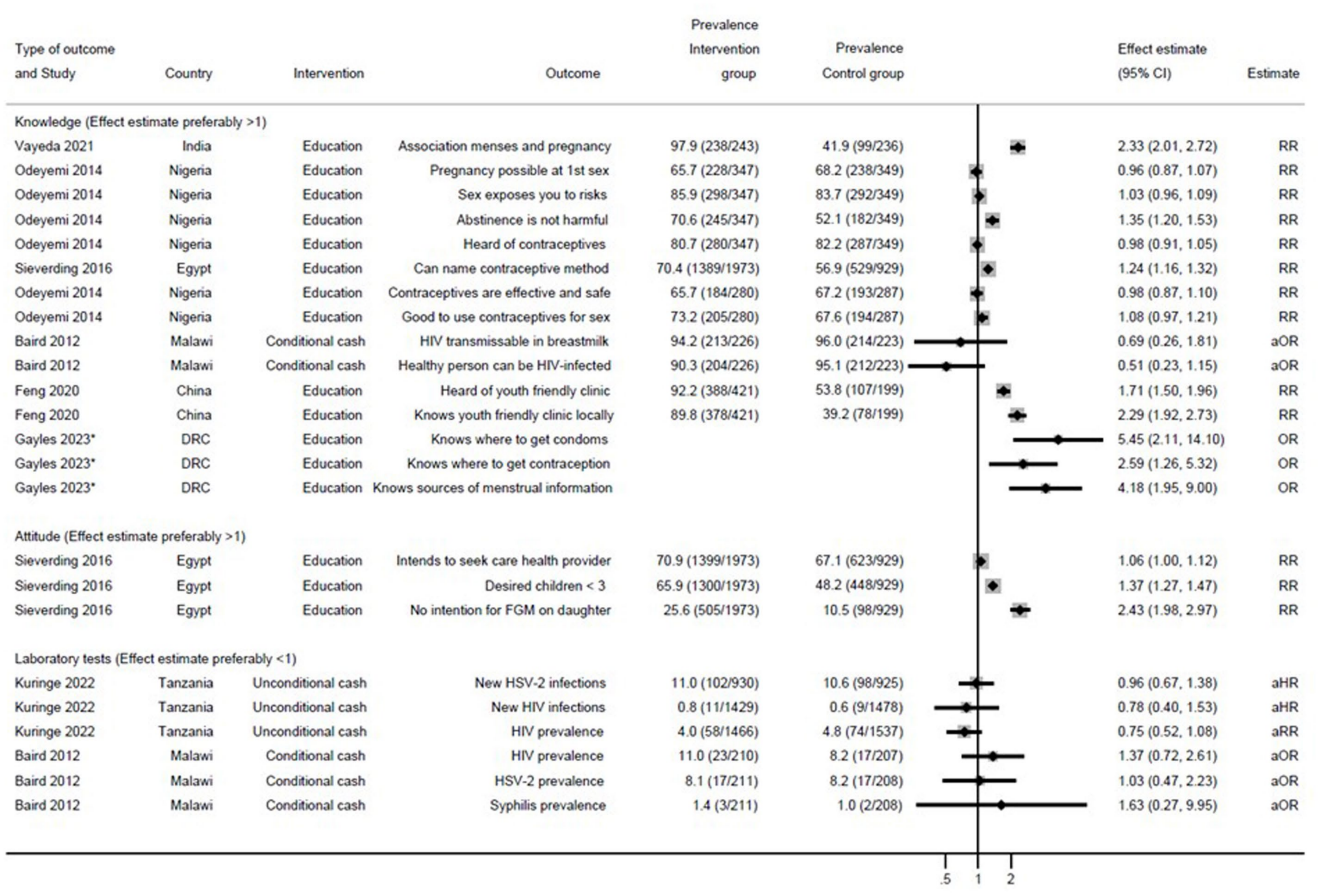
Effect of interventions on knowledge, attitude, and laboratory test outcomes among out-of-school girls. RR, risk ratio. aOR, adjusted odds ratio. aHR, adjusted hazard ratio. FGM, female genital mutilation. DRC, Democratic Republic of the Congo. Because of the lack of uniformity in outcomes, results could not be pooled. The forest plot is only used to summarize results.*Gayles et al. (29): This study did not provide prevalence.

Six studies evaluated the effect of an intervention on the SRMH knowledge of out-of-school girls (Figure 2). Following an SRMH educational intervention in India, girls were reported to have greater knowledge on the association between menses and pregnancy than before the intervention began (RR 2.33, 2.01–2.72) (27). In an educational SRMH intervention in Nigeria, more girls in the intervention arm knew abstinence was not harmful (RR 1.35, 1.20–1.53) compared to the control arm (23). In a multisectoral intervention in China, more girls in the intervention area knew about youth-friendly clinics compared with girls in the control area (Figure 2) (28). A large effect of multiple educative sessions spread over 9 months was seen on knowledge about condoms, contraception and menstrual information in the Democratic Republic of the Congo with ORs of 2.6 and higher (Figure 2); unfortunately, prevalences of these outcomes were not available by study arm (29). Girls’ attitudes toward reproduction, healthcare, and female genital mutilation were evaluated in Egypt after an educational intervention (22). This study reported that a significantly higher proportion of girls in the intervention arm reported they would not use female genital mutilation if they ever had daughters (RR 2.43, 1.98–2.97), and planned to have fewer than three children (RR 1.37, 1.27–1.47) compared with girls in the control arm (22).

### 3.5 Effect of interventions on sexual and reproductive health behavior

**Figure 3:**
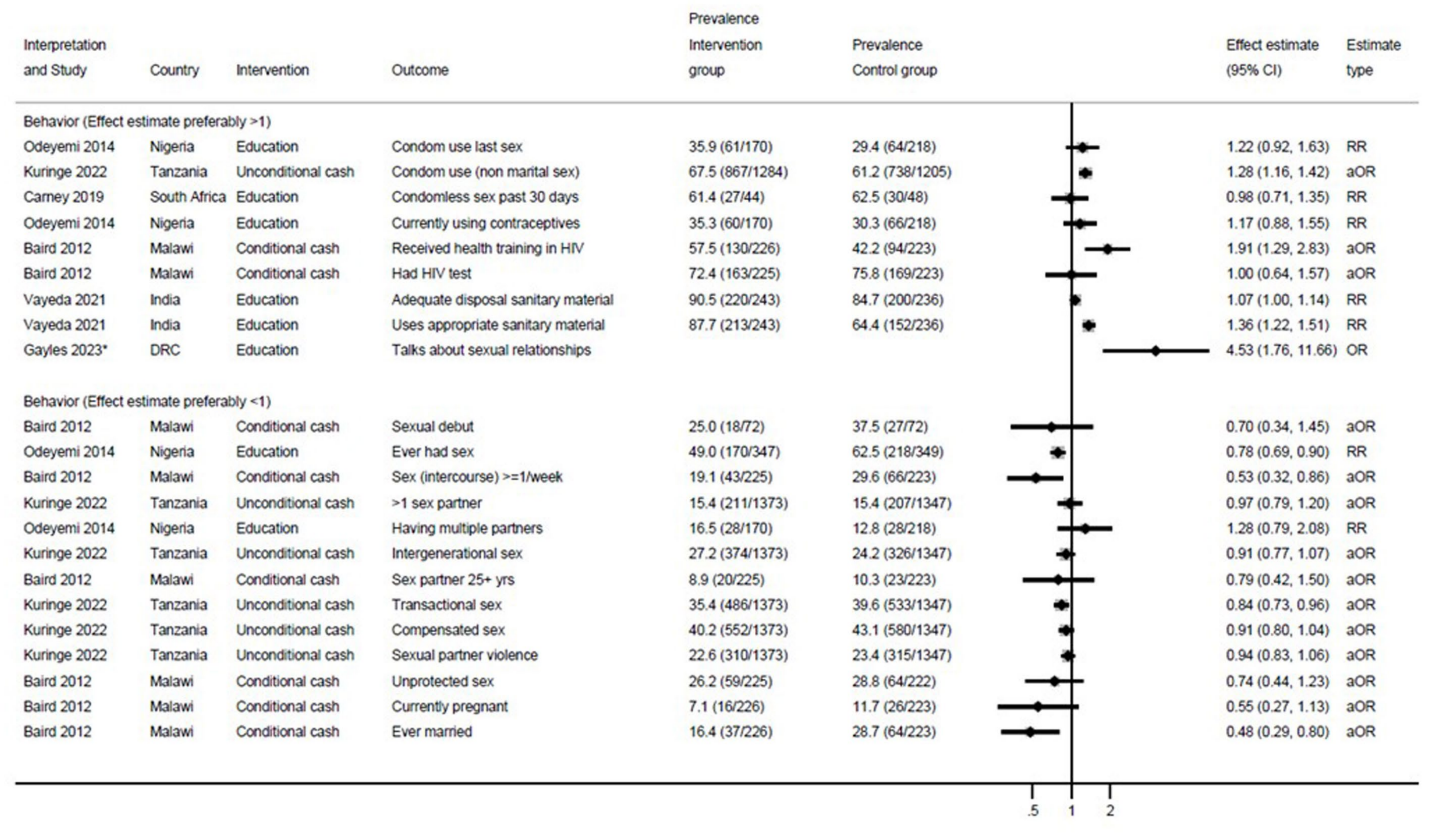
Effect of interventions on behavior outcomes among out-of-school girls. RR, risk ratio. aOR, adjusted odds ratio. DRC, Democratic Republic of the Congo. Because of the lack of uniformity in outcomes, results could not be pooled. The forest plot is only used to summarize results. *Gayles et al. (29): This study did not provide prevalence.

Five studies compared health-related behaviors between the control and intervention groups. Of these, five reported on the effectiveness of at least one behavioral outcome (Figure 3). In Tanzania, girls receiving unconditional cash self-reported higher condom use (aOR 1.28, 95% confidence interval [CI] 1.16–1.42) compared to girls in the control group (25). In Malawi, among girls receiving conditional cash transfer based on school attendance a higher proportion received health training in HIV (aOR 1.91, 95% CI 1.29–2.83), compared with those not receiving conditional cash (26). One study in India which provided education on menstrual health to out-of-school girls reported an improvement in menstrual outcomes such as use of appropriate sanitary material (RR 1.07, 1.00–1.14) and adequate disposal of used products (RR 1.36, 1.22–1.51) over time, when comparing baseline surveys with those conducted at the end of the study (27). Compared to girls in the control group, girls who had participated in the SRH training reported more frequent discussion of sexual relationships with other persons in a study in the Democratic Republic of the Congo (OR 4.53, 1.76–11.66) (29). A 6-month education programme in Nigeria noted a decrease in self-reported sexual activity (RR 0.78, 0.69–0.90) among girls in the intervention group compared to the control arm (23). A study in Malawi using cash conditioned on school return saw a decrease in self-reported sexual activity (aOR 0.53, 0.32–0.86, respectively) among girls in the intervention group compared to the control arm (26). They additionally reported that marriage was less frequent among girls in the conditional cash arm (aOR 0.48, 0.29–0.80) compared to the control group (26).

### 3.6 Effect of sexual and reproductive health interventions on laboratory tests of HIV, HSV-2 and syphilis

Two trials included testing for HIV and HSV-2 in their evaluation. One trial was conducted in Tanzania and used unconditional cash following an SRH education programme (25). No effect was seen on HSV-2 (11.0% in intervention and 10.6% in control arm, respectively) or HIV (4.0 and 4.8%, respectively; Figure 2). A trial in Malawi used cash conditional on attending school among out-of-school girls in the intervention arm (26). Again, no effect was seen on HSV-2 (8.1 and 8.2%, respectively) or HIV (11.0% vs. 8.2%, respectively). Among out-of-school girls, no effect of the intervention was seen on the prevalence of syphilis (2 and 1%, respectively). Among in-school girls, prevalence of syphilis was low (0.2 and 0.5%, respectively).

### 3.7 Qualitative components of included studies

Two studies included qualitive components to evaluate project acceptability and behavior change. One study in the Democratic Republic of the Congo, using in-depth interviews, noted that household responsibilities could be an occasional obstacle to participation among out-of-school girls (29). Participants in another study (South Africa, *n* = 21, four focus group discussions) thought the intervention as helpful and increasing their knowledge and skills. However, some participants reported difficulties in making changes because of persistent surrounding stressors (e.g., boyfriends, lack of access to health and social services). In addition, participants noted that they needed more time to implement change and practice skills (24).

## 4 Discussion

Girls who are not attending school or have left school prematurely are at higher risk of SRMH harms compared with their school-going peers and would benefit from programmes supporting their health needs. We identified just eight studies about programmes for out-of-school girls with an SRMH component, ranging from mainly educative programmes to programmes with a cash component. Outcomes reported were diverse, with some positive effects noted on knowledge, attitude, and behavior, but not on laboratory tests. Study quality was moderate-to-low, and identification of out-of-school girls was likely not random or systematic in three of the seven studies.

Once girls are out of school, they can become invisible and isolated from or within society. Their isolation makes them vulnerable to mental health issues. A Tanzanian study among out-of-school girls noted that about one-third of girls had depressive and anxiety symptoms, while another among out-of-school mothers in Sweden reported a higher chance of postpartum depression compared to schooling mothers (24, 25). Moreover, out-of-school girls isolation makes them harder to recruit for research studies and programmes. An illustration of such recruitment difficulties is evident in vaccination programmes for human papilloma virus (HPV; Supplementary Table S7), where vaccination coverage is reported to be lower among out-of-school girls compared to schoolgirls despite their higher risk of HPV exposure and considerable efforts made by facility-based outreach programmes to increase uptake among out-of-school girls (13).

Within the small number of interventional studies that were identified, there was a substantial diversity in the types of programmes evaluated and outcomes assessed, and this may be a consequence of the different populations, settings, and backgrounds present in the different countries examined. Education was the main component of the intervention in four of the seven included studies, and an important component of the evaluation of the intervention; improvement of knowledge and a healthier attitude was reported by three studies. Increase in knowledge may be affected by the time between education and evaluation, with shorter time periods potentially resulting in better recollection (22–24, 27). Additionally, responses may be affected by type of data collection (face-to-face interviews versus self-interviews) and social desirability bias (30). Although knowledge is a basic requirement, it may not be sufficient to achieve healthy behavior (31). Behavior that protects from harm is harder to achieve but may be more important as an outcome of an intervention than change in knowledge or attitude (32). Cash programmes reported desirable behavior for some outcomes in two low-income countries, and relatively more than educational programmes (five of the eight outcomes where *p* < 0.05). In Malawi, the cash programme was conditional on out-of-school girls returning to school, whereas in Tanzania, cash was provided on top of 10 h-long sessions of social and behavior change communication (25, 26). Both programmes may have been able to provide both the knowledge and means for behavior change; the authors suggest that the cash may have helped the girls to reduce risky behavior. However, specific to the private nature of SRMH, responses to questions on SRMH rely on self-report and are hard to verify. Laboratory testing for HIV and HSV-2 in some African settings may be used as an indicator of harm from behavioral exposure; however, the two cash studies which used laboratory tests did not show differences in HIV or HSV-2 comparing intervention to control groups of out-of-school girls. Baird et al. (26) did however note a protective effect of their programme on HIV infections among schoolgirls in the same study and reported that the study only had sufficient power to detect very large effects on HIV among out-of-school girls. Kuringe et al. (25) noted a protective effect of the programme on HSV-2 conversion in a rural subpopulation at low risk of HIV (25, 26), but postulated that overall, the cash amount provided may not have been sufficient to bring young girls out of the risk behavior. Although there are other objective measures which can identify recent vaginal exposure to semen, their use may be limited for the evaluation of impacts of out-of-school programmes on sexual risk behavior (30).

This review has several limitations. Although the search identified some studies of interest, most studies could not be included because they provided insufficient details (Supplementary Table S4). A common reason for exclusion was that studies included in-school and out-of-school girls, but the results were not stratified by school attendance status, preventing evaluation. A definition of out-of-school youth was regularly lacking (five of eight studies), and definitions used in three studies were not uniform. Evaluations in the short term may not reflect sustained changes; two studies evaluated outcomes within 1 and 6 months (23, 24). However, in the absence of a sexual relationship, it may not be clear how the acquired knowledge would have been put to practice and would be durable. We only assessed quantitative studies in this review, so have no information on how participants experienced the programme in exclusive qualitative studies. Furthermore, interventions were commonly adapted to the local context, which is important, but makes it harder to assess and compare components which may be generalisable to other settings. Similarly, due to the lack of uniformity in study outcomes, results could not be pooled. The studies included in this review (Supplementary Tables S3, S6) used several methods to identify and recruit participants, varying from door-to-door screening of households, use of workplace locations with a high likelihood of the presence of out-of-school girls, to inclusion of peer outreach and youth centers. These (necessary) approaches increase the cost of interventions for out-of-school, compared with in-school adolescents, while recruitment through workplace locations and youth centers may increase the risk of selection bias in the outcome measured. Several studies commented how much harder it is to recruit out-of-school compared with in-school adolescents and, that out-of-school populations risk being convenience samples than a sample representative of the population (28). Surveys pre-and post-implementation of an intervention are at risk of being influenced by factors related to time which is important for outcomes such as first sexual exposure; however, only one study used a before-after design and this study focused on menstrual health, where the effect of time is not likely to have affected the outcome (27).

The importance of programmes for out-of-school girls has been recognized: e.g., the United Nations Population Fund (UNFPA) developed guidelines for out-of-school comprehensive sexuality education (18). However, we only identified eight studies in low-and middle-income countries for this review that could be evaluated for SRMH outcomes and 11 studies which could not be evaluated but were (partly) targeting out-of-school girls (Supplementary Table S4); no study focused on out-of-school girls’ mental health was identified. To only identify eight studies using a systematic approach illustrates the urgency for further studies to improve conditions among this vulnerable group. Ideally, programmes should be developed to retain girls in school: A recent scoping review noted 18 studies which evaluated interventions to keep girls in school, varying from provision of funding, school-based interventions for learners, community-based interventions (such as youth centers, media exposure) and education system interventions (such as school lunches, teacher awareness) (33). The authors of the review report that provision of school fees and other school supplies (including menstrual items) can reduce school dropouts, and increase enrolments and school attendance; however, no numerical results were presented. The effectiveness of the interventions are therefore unclear. For girls who cannot or will not return to school, programmes are needed to ensure they have the knowledge, life skills and support needed to reduce their vulnerabilities, improve their network, and develop healthy behaviors. An example of such a programme is the DREAMS study (Determined, Resilient, Empowered, AIDS-free, Mentored, and Safe), and a qualitative study by Kuringe et al. (25) illustrated beneficial effects on reported condom use and transactional sex (25, 34). Additional programmes for out-of-school youth without a formal evaluation have been described (19, 35): e.g. on its website, UNFPA reports on 14 country case studies (18, 36). A study in Kenya reported on the integration of youth mental health into health and life-skill safe spaces for out-of-school adolescents (37). In India, studies are ongoing into the use of artificial intelligence (chatbots) to improve SRMH knowledge, which could be useful for any adolescent, but particularly for the out-of-school group (38). These initiatives are encouraging, but formal evaluations of their effectiveness would be helpful as an evidence-base to support programmes in other settings.

## 5 Conclusion

Evaluations of interventions to improve the health and wellbeing, specifically related to SRMH, of out-of-school girls are lacking. Studies published are limited in type of intervention, and measures of outcomes, with the majority restricted to sexual education and adapted to local conditions (40). Furthermore, few interventions have considered out-of-school girls broader health and wellbeing needs, such as community and peer group support, which have potential to reduce girls’ isolation and stigma. Cash transfer programmes seem promising in providing access to education and supporting adolescents with crucial financial means. Targeted interventions, incorporating both cash transfer and comprehensive sexual education elements for out-of-school girls may prove beneficial. However, to be able to formulate effective policy targeting out-of-school girls, it is recommended that future studies of sufficient duration, sufficient resources, and high methodological quality measure the effect of interventions on a variety of outcomes, including laboratory confirmed STI incidence, behavioral change, and mental health.
