# Supplementary material for: Interventions aiming to improve menstrual, sexual, reproductive, and mental health among out-of-school girls: a systematic review
Source: Front Public Health. 2024 Dec 4;12:1440930. doi: 10.3389/fpubh.2024.1440930 (PMC11656019; doi:10.3389/fpubh.2024.1440930)
Supplement: Supplementary file 1 [file Data_Sheet_1.pdf]

Supplementary Material

Interventions aimed at improving menstrual, sexual, reproductive, and mental health among out-of-school girls: a systematic review

Karinn Farquharson [1], Alexandra Quinn-Savory [1], Garazi Zulaika [1], Linda Mason [1], Susan Nungo [1], Elizabeth Nyothach [2], Holger Unger [1, 3, 4], Muthusamy Sivakami [5], Philip Spinhoven [6], Penelope A. Phillips-Howard [1], Anna Maria van Eijk [1*]

*** Correspondence:**
Corresponding Author Anna Maria van Eijk, Department of Clinical Sciences, Liverpool School of Tropical Medicine, Pembroke Place, Liverpool, L3 5QA, United Kingdom
anna.vaneijk@lstmed.ac.uk

Contents

Search strategy ... 2
Table S1 Search terms used by database ... 2
Table S2. PICOTS Framework ... 3
Figure S1 Quality assessment of included studies ... 4
Table S3. Recruitment of out-of-school girls in included studies and definitions used ... 5
Table S4. Interventions used in eleven excluded studies which reported to include out-of-school girls ... 6
Table S5. Description of intervention of not-included studies which included out-of-school girls ... 9
Table S6. Recruitment of out-of-school girls in excluded studies which included out-of-school girls ... 11
Table S7. Reaching out-of-school girls for Human Papilloma Virus vaccination ... 12

Search strategy

Terms were searched with 'OR' and results were combined with 'AND' to create an extensive list of relevant studies.

Table S1 Search terms used by database

**Area**	**Search Terms PubMed**	**Web of Science**	**Embase, Medline, Global Health, Psycinfo, Cinahl**
Out-of-School	out-of-school OR "out of school" OR school-drop-out OR "school drop out" OR "school drop-out" OR non-school*	out-of-school OR "out of school" OR school-drop-out OR "school drop out" OR "school drop-out" OR non-school*	out-of-school OR out of school OR school-drop-out OR school drop out OR school drop-out OR non-school
Adolescent Girls	adolescen* OR teen* OR girl* OR female*	adolescen* OR teen* OR girl* OR female*	adolescent OR teen OR girl OR female
Intervention	Intervention OR trial OR program* OR cohort	Intervention OR trial OR program* OR cohort	Intervention OR trial OR program OR cohort OR programme
Sexual and Reproductive Health	"sexual health" OR "reproductive health" OR menstr* OR menses	"sexual health" OR "reproductive health" OR menstr* OR menses	sexual health OR reproductive health OR menstruation OR menses OR menstrual
Mental Health	depress* OR anxi* OR quality of life OR quality-of-life OR well-being OR "stress, psychological" OR stress* OR distress OR "mental health"	depress* OR anxi* OR "quality of life" OR quality-of-life OR well-being OR "stress, psychological" OR stress* OR distress OR "mental health"	Depression OR anxiety OR quality of life OR quality-of-life OR well-being OR stress, psychological OR stress OR distress OR mental health

Table S2. PICOTS Framework

Components	Characteristics
**Population**	Adolescence is generally specified as 10 – 19 years old. However, definitions may differ by study and each study's own description of adolescence will be used. The definition for out-of-school will be used as described in the studies.
**Intervention**	Interventions must aim to improve adolescent menstrual, sexual, reproductive or mental health in some way. Any interventional study will be included such as randomised and non-randomised controlled trials, analyses/evaluations of programmes and any 'before and after' experiment using a cohort study or repeated surveys. Quantitative studies will be used.
**Comparison**	Comparisons made will be specific to the study e.g., intervention groups may be compared to a control group of out-of-school girls, or schoolgirls who also received the intervention or before/after comparisons, whereby out-of-school girls are their own controls.
**Outcomes**	Because of the anticipated limited evaluations among out-of-school girls, we will not prespecify outcomes; they will be categorised into menstrual, sexual, reproductive, and mental health outcomes. Examples of menstrual outcomes include use of menstrual hygiene product, type of item used, reduction of genital infections related to menstrual hygiene product, and general knowledge of menstrual cycle. Examples of SRH outcomes include use of condoms, family planning methods, pregnancy, genito-urinary infections, HIV infection and general knowledge of SRH issues. Examples of mental health issues include conditions such as depression, or anxiety.
**Timing**	No time restrictions
**Setting**	Studies from any country will be considered for the review. For studies published in another language than English, an attempt will be done to identify a colleague who can help with translation. If no colleagues can be found for a specific language, the study will be excluded. There will be no restriction for continent.

Figure S1 Quality assessment of included studies

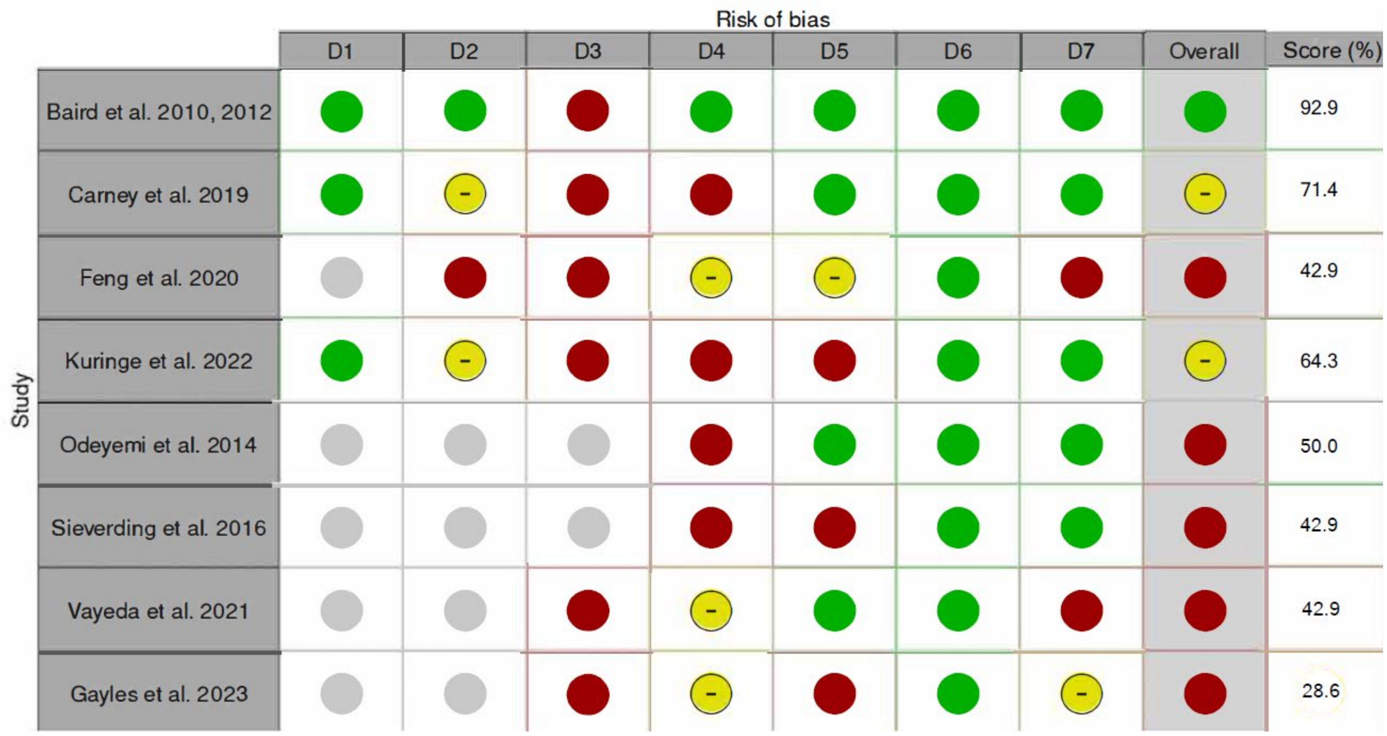

D1: Random sequence generation (selection bias)
D2: Allocation concealment (selection bias)
D3: Blinding of participants and personal (performance bias)
D4: Blinding of outcome assessment (detection bias)
D5: Incomplete outcome data (attrition bias)
D6: Selective outcome reporting (reporting bias)
D7: Other bias

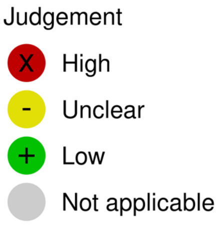

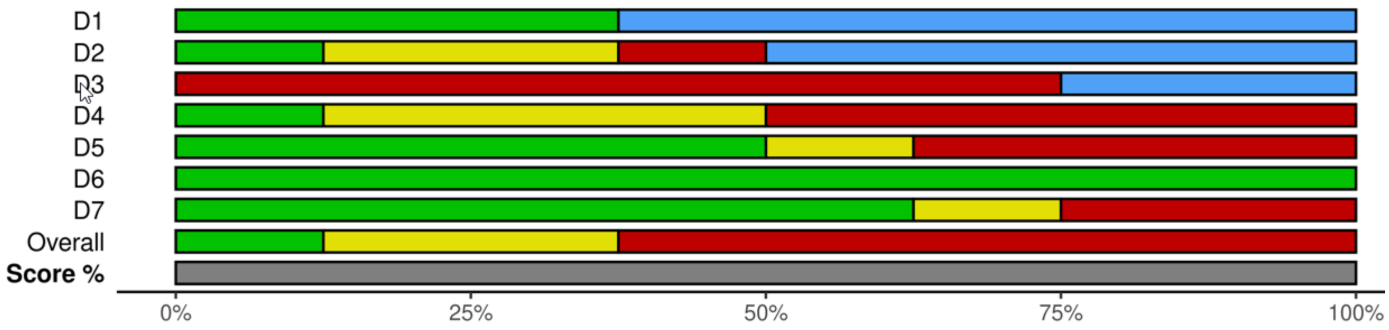

Summary plot

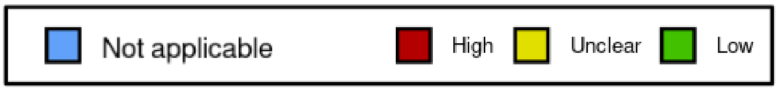

Scoring system: 2 points for low risk of bias, 1 for high risk of bias and 0 for unclear or not applicable. Total of 14 points possible. **Scoring**: -Good quality: 80% or more; -Moderate quality: 51-79%; -Low: ≤50%

Robvis: McGuinness LA, Higgins JPT. Risk-of-bias VISualization (robvis): An R package and Shiny web app for visualizing risk-of-bias assessments. Research Synthesis Methods. 2020

Table S3. Recruitment of out-of-school girls in included studies and definitions used

Study	Country	Definition of out-of-school population	Recruitment of out-of-school population
Baird et al. (2010, 2012) (25, 39)	Malawi	Those not enrolled in school at baseline	Census estimates, door-to-door to screening for eligibility
Carney *et al.* (2019) (23)	South Africa	"Dropped out of school"	Use of young female outreach workers to meet potential participants on the streets and in other areas where the girls were known to frequent
Feng *et al.* (2020) (27)	China	Definition not reported.	Unmarried young people aged 12–24 years from randomly selected communities or worksites in the investigated counties were included
Kuringe *et al.* (2022) (24)	Tanzania	Either never enrolled or have dropped out of school for at least a month at the time of study enrolment	Household survey among young women in the identified villages to determine young women who were out-of-school
Odeyemi *et al.* (2014) (22)	Nigeria	Never been to school or stopped school attendance	Randomly selecting a sample of clusters from the two markets and interviewing all eligible girls attached to stalls from these clusters
Sieverding *et al.* (2016) (21)	Egypt	Definition not reported	Word of mouth, public announcements, events at local youth centres and meetings with the girls' parents
Vayeda *et al.* (2021) (26)	India	Definition not reported	Adolescent girls of selected villages were enlisted
Gayles *et al.* (2023) (28)	Democratic Republic of Congo	Definition not reported	Out-of-school very young adolescents attending Growing Up GREAT! clubs in the same neighbourhoods as pre-selected schools in addition to a random sample of adolescents selected from a listing of households known to contain out-of-school adolescents

Incentives such as snacks and food rations were used to increase enrolment and decrease attrition rates.

Table S4. Interventions used in eleven excluded studies which reported to include out-of-school girls

Author	Country, study period	Design	Population	Age, years, mean (SD)	Intervention*	Comparison	Sample size	Outcome measures and results	Reason excluded
**Aninanya 2015 (1)**	Ghana, 2005-2008	Intervention vs. control communities, cluster randomized trial	In- and out-of-school youth	Adolescents 15-17 years at enrolment. Mean age 16 years, no sd presented<br>Female: 51.4%<br>Female and out-of-school: 3.8%	Social learning intervention, implementation 2005, evaluation 2008	Community mobilisation and youth-friendly health services training only	26 communities, cohort of 2664 adolescents	Use of STI services (aOR 2.47, 1.78-3.42)<br>HIV counselling and testing (aOR 1.16, 0.85-1.58)<br>Perinatal services (1.89, 1.37-2.60) and service satisfaction	Outcome not stratified by gender and school status, small sample size for out-of-school population
**Austrian 2021 (2)**	Kenya, 2015-2017	Pre- and post-intervention survey, Randomized trial	In- and out-of schoolgirls	Mean age: 12.6 years (Kibera) And 11.9 years (Wajir), no sd presented<br>Female: 100%<br>Out-of school Kibera: range 0.7-1.4%; Wajir range 16.6-27.2%	Intervention packages consisting of violence prevention only, violence prevention + education, violence prevention + education + health, violence prevention + education + health + wealth creation	No control group: packages compared	Wajir: 80 clusters, n=2297<br>Kibera: n=3296	Kibera: education conditional cash transfer had small effects on grade attainment but larger impacts on completion of primary school, the health intervention improved SRH knowledge and condom self-efficacy, and the wealth intervention improved financial literacy and savings behaviour.<br><br>Wajir: education conditional cash transfer increased school enrolment and grade attainment, the wealth intervention improved savings behaviour.	Outcomes not stratified by school status
**Bhatia 2023 (3)**	India, 2016-2020	Parallel-group, two-arm, superiority, cluster-randomised controlled trial	In- and out-of-school adolescent girls	10-19 years<br>57% 10-14 years<br>Female: 100%<br>Out of school: NR	Community youth team delivering participatory adolescent groups, youth leadership activities and livelihood promotion	Only livelihood promotion offered to the control arm	38 clusters, cohort at baseline n: 3324<br>Endline: 1478	-School attendance (aOR 1.39, 95% CI: 0.89, 2.16)<br>-Dietary diversity (aDiff 0.34, 95% CI: -0.23, 0.92)<br>-Mental health problems utilising BPM-Y (aDiff: 0.02, 95% CI: -0.06, 0.12)	-No separate information for out-of-school girls
**Firestone 2016 (4)**	Liberia, 2014	Intervention and control sites, cluster randomized trial	Out-of-school young adults	15-35 years, median age 29 years<br>Female: 69.1%<br>Out-of-school: 100%	6-day intensive basic SRH education, implementation Jan-Mar 2014, evaluation Jun-Jul 2014	No 6-day intensive course on SRH	34 sites to treatment, 26 to control<br>Baseline n=1255, endline n=1142	Always Condom use: regular partner, always uses condom RR (raw data endline) 1.73, 1.00-2.98. Casual partner RR (raw data endline) 1.17, 0.81-1.70<br>Contraceptive use (women only, modern method): RR (raw data endline) 1.20, 1.04-1.40<br>HIV testing and counselling: RR (raw data endline) 2.09, 1.88-2.33	Age-group too broad and no stratification by age
**Gourlay 2019 (5)**	Kenya and South-Africa	Pre- and post-intervention surveys	In- and out-of-school AGYW	10-22 years	Multi-sectoral intervention	Engagement of participants from all three sites were compared		-Awareness of DREAMS initiative higher among younger women	-Insufficient detail in reporting; outcomes not stratified by school status

**Lou 2004 (6)**	China, 2000-2001	Intervention and control site, pre-and post, in two comparable towns	Unmarried young adults	15-24 years<br>27% <17 years<br>Female: 42.8%<br>Out of school 58.6%	Educational SRH programme. Evaluation after 20 months of implementation	Standard programme and services	Intervention: n=1220<br>Control: n=1007	Ever contraceptive use: intervention 98.9% vs. 82.8% in control at endline<br>Regular contraceptive use: 89.0% vs. 44.6%<br>Ever condom use: 97.4% vs. 81.2%<br>Contraceptive use at onset of sexual intercourse: 79.4% vs. 69.0%	Outcome not stratified by school status
**Mehra 2018 (7)**	India, 8 rural sites, 2009-2013	Pre- and post-intervention surveys	Young persons	10-24 years, 43% 10-14<br>28% 15-19<br>Female 53.3<br>27% out of school	Youth information centres	No clear control group	1770 (only endline)	Can't be evaluated reliably<br>-Early marriage<br>-Early pregnancy<br>-School retention	Outcome not stratified by school status, age group<br>Design not clear: no results from baseline, and numbers drop from 1770 in demographics table to < 200 in tables describing effect on outcomes. There seems to be low use of intervention
**Munea 2020 (8)**	Ethiopia, 2018-2018	Survey in intervention and control areas	Unmarried adolescents, within 5 km radius of health facility	15-19 years,<br>42% 15-17 years<br>Mean 17.6 (1.4)<br>Female 44.4%<br>34% out of school	Youth friendly services programmes in health facility	Areas around clinics without youth friendly service programmes	545 from programme areas, 580 from non-programme areas	-Risky sexual behaviour intervention 25% vs. 29% control, p=0.12<br>"A point increase in parent–adolescent communication score reduced risky sexual behavior by 20% (AOR 0.80, 95% CI 0.75–0.85)."<br>-out-of-school risk factor for risky sexual behaviour, aOR 1.64, 1.12-2.39	Outcome not stratified by school status
**Oberth 2021 (9)**	Zimbabwe, 2013-2019	Programme evaluation	In- and out-of-school AGYW	10-24 years<br>48% 10-14 years<br>43% 15-19 years<br>Female: 100%<br>Out of school: 17.6%	Structured peer group intervention	No clear control group	Graduates: 58471 (completed 30 of 40 exercises)<br>Non-graduates: 33141	Graduates vs. non-graduates<br>-take HIV test OR 2.78, 2.52-3.10<br>-get married OR 0.63, 0.55-0.73<br>-drop out of school OR 0.60, 0.53-0.69<br>-reporting sexual abuse OR 0.92, 0.67-1.25<br>-reporting use of family planning OR 0.95, 0.87-1.04<br>-pregnancy OR 1.05, 0.88-1.24	-Comparison between graduates and non-graduates<br>-No separate information for out-of-school girls
**Patel 2018 (10)**	India, 2013-2017	Pre-and post-intervention surveys and comparison area	Adolescents, in and out of school	15-18 years, out of school, girls 50% (11-18 years for school-based)	Capacity building workers, local leaders. Health days.	Intervention area compared with control area using before/after surveys in both areas	254 & 256 at baseline, and 318 & 321 at endline for intervention and control block	Among girls: At baseline intervention areas higher % for most indicators. In intervention areas:<br>-Awareness STI: 3.6%† increase post intervention<br>-Awareness HIV/AIDS: 2.7% increase<br>-Knowledge contraception: 2.1% increase<br>-use of sanitary napkin: 4.6% higher<br>-Practice of reusing absorbent: decrease 8.6%	-outcomes not separated by school status

**Tandon 2012 (11)**	USA, 2007-2008 Low-income area	Pre- and post-intervention surveys	Out-of-school youth	16-24 years, mean 18.8, sd 2, n=135, Female 54.4%	Mental health intervention	A pre-post-test assessment at baseline and 12-month follow-up	136	-Intervention had no effect on depressive symptoms or coping strategies<br>-Among participants with depression Increase in use of mental health service from 24% (n=51) to 31% (n=42)	-outcomes not stratified by age-group and gender

AGYW: adolescent girls and young women. SRH: sexual and reproductive health. Sd: standard deviation. aOR: adjusted odds ratio.

*For further description see table S4

†Percentage point, e.g., 29.2% before and 32.8% after intervention results in 3.6% increase

Table S5. Description of intervention of not-included studies which included out-of-school girls

Study	Country	Description of intervention
Aninanya 2015 (1)	Ghana	Community mobilisation, health worker training in youth friendly health services approaches, school based sexual health education, out-of-school peer outreach to improve self-efficacy and interaction between young people and health workers.
Austrian 2021 (2)	Kenya	AGI-K intervention consisting of nested combinations of four single-sector interventions (violence prevention, education, health and wealth creation).
Bhatia 2023 (3)	India	JIAH intervention delivered by community youth team consisting of friends of youth, youth leadership facilitators and livelihood promoters. Teams conducted: peer-led participatory learning and action meetings to mobilise changes for adolescent health and development; group-based youth leadership activities to build adolescents confidents and resilience; and livelihood promotion with adolescents and their families to provide training and practical skills.
Firestone 2016 (4)	Liberia	6-day intensive course on SRH and life skills with participants split in two teams. On Day 2 topics included puberty, sex, and reproductive health; Day 3: HIV/AIDS; Day 4: contraception; Day 5: communication skills/taking action; Day 6: celebration. The interactive programme included games, trivia, small group discussions, role playing and body mapping.
Gourlay 2019 (5)	Kenya and South-Africa	Core package consisting of evidence-based intervention to enhance individual agency to access HIV prevention and SRH services. Interventions included condom promotion and provision, PrEP, post-violence care, including PEP, HIV testing services, expand and improve access to voluntary family planning services, social asset building, parenting programmes, educational subsidy for transition to and attendance of secondary school, combination socio-economic approaches and community mobilisation.
Lou 2004 (6)	China	Activities on building awareness, information brochures on sexual health, educational videos, lecture on sexual health, group activities on STI prevention, sexual communication and negotiation, counselling, contraceptives
Mehra 2018 (7)	India	Age and culturally appropriate life skill-based educational sessions, focusing on SRH and rights. The national curriculum 'Life Skills & Adolescent Education Programme' was adapted to strengthen the components on early marriage and early pregnancy to suit the objectives of the intervention. The curriculum had additional activities with the understanding to promote education retention as a pathway to delay the age of marriage and first pregnancy. Further components included the use of peer educators, development of posters and activities at the family and community level. Two days of training was followed by refresher sessions of the functionaries in the relevant government departments on child marriage and SRH.
Munea 2020 (8)	Ethiopia	Government run youth friendly service programme, including the implementation of an age driven approach in existing public health facilities. Moreover, peer educators provide community- and school-based SRH information by providing more emphasis on reduction of early sexual initiation, unsafe sex, and its complications, and promotion of SRH-service utilization (implemented since 2006).
Oberth 2021 (9)	Zimbabwe	Sista2Sista girls-only clubs: safe spaces for support and mentoring conducted by age groups. This consisted of weekly meetings over the course of one year, following 40-exercise curriculum, guided by club mentor. The different exercises focus on topics such as self-awareness, communication, gender and power, family planning, sexually transmitted infections, HIV, stigma and discrimination, menstrual health, cancer awareness, sexual and gender-based violence, traditional and cultural practices, consent, and financial awareness, among others.
Patel 2018 (10)	India	Intervention focussed on sexual and reproductive health, nutrition, and substance abuse. Capacity building of frontline workers and local level committees. Adolescent health days in intervention villages with referrals to adolescent-friendly health clinics for counselling and clinical services.

Tandon 2012 (11)	USA	Mental health clinician at an employment training programme in youth opportunity center, a peer-led depression prevention intervention, and training sessions for employment training staff

Abbreviations: AGI-K: Adolescent Girls Initiatve-Kenya; HIV: human immunodeficiency virus; JIAH: Jharkhand Initiative for Adolescent Health; PEP: post-exposure prophylaxis; PrEP: pre-exposure prophylaxis; SRH: sexual and reproductive health; STI: sexually transmitted infection.

Table S6. Recruitment of out-of-school girls in excluded studies which included out-of-school girls

Study	Country	Recruitment of out-of-school population
Aninanya 2015 (1)	Ghana	Peer outreach: participants were selected through random sampling of district compounds from the NHDSS database in 2005 and retargeted for interview in 2008.
Austrian 2021 (2)	Kenya	Rapid household listing conducted in each cluster.
Bhatia 2023 (3)	India	Door-to-door visits in each cluster (a purposively selected geographic area) to identify eligible girls and seek individual consent (including consent from the caregiver if aged <18 years old).
Firestone 2016 (4)	Liberia	Eligible study participants were current USAID Advancing Youth Project learners between the ages of 15–35. They were randomly sampled from the group of learners available on the day that the data collection team visited a selected learning site.
Gourlay 2019 (5)	Kenya and South-Africa	Door-to-door visits followed by enrolment interviews.
Lou 2004 (6)	China	Unclear: "All unmarried youths (15–24 years), irrespective of whether or not they were in school or college, and who would not marry and leave two towns within one year, were recruited in the study".
Mehra 2018 (7)	India	Peer outreach. Random community sample for surveys.
Munea 2020 (8)	Ethiopia	Peer outreach and youth friendly clinics. Household listings for survey.
Oberth 2021 (9)	Zimbabwe	Census estimates, door-to-door to screening for eligibility (risk assessment tool to help determine if girls are at high risk in five key areas: self-awareness, education, social relationships, sexual knowledge and financial awareness).
Patel 2018 (10)	India	Not reported
Tandon 2012 (11)	USA	Employment training programme in youth opportunity centre

Abbreviations: NGO: Non-Governmental Organisation; NHDSS: Navrongo Health and Demographic Surveillance System; USAID: United States Agency for International Development.

Table S7. Reaching out-of-school girls for Human Papilloma Virus vaccination

		Population	Out-of-school girls	Schoolgirls
Riviere 2021 (12)	Haiti, Port-au-Prince 2016-2017	Girls 9-14 years, clinic-based approach for OOS, vs. school-based approach	1307/1698 (77.0%) 1st dose<br>1199/1698 (70.6%) 2nd dose	School based for schoolgirls<br>687/747 (92.0%) 1st dose<br>673/747 (90.1%) 2nd dose
Isabirye 2020 (13)	Uganda, 2016 DHS data	Girls 10-14 years	93/615 (15.1) any dose	Schoolgirls<br>1244/5478 (22.7) any dose
Msyamboza 2017 (14)	Malawi, Zomba & Rumphi districts	Girls 9-13 years, clinic-based approach, identified by community health workers	403/765 fully vaccinated (52.7%)	Schoolgirls in standard 4<br>10,070/11240 (89.6%)
Gallagher 2017 (15)	Review HPV vaccination strategies	"Out-of-school girls. National primary school enrolment ratios indicate the proportion of girls out-of-school was 5% or less in 23% of the countries with data (10/43), between 6% and 20% in 56% of countries (24/43), and over 20% in nine countries (range 23–38%). Almost a third of experiences (27%) had no reported strategy for reaching out-of-school girls, another third (35%) relied on them attending health facilities for vaccination and the remaining experiences used outreach. Outreach was used in all nine countries with poor school enrolment and reportedly increased coverage."		

References Supplement

1. Aninanya GA, Debpuur CY, Awine T, Williams JE, Hodgson A, Howard N. Effects of an adolescent sexual and reproductive health intervention on health service usage by young people in northern Ghana: a community-randomised trial. PLoS One;10(4):e0125267. doi:10.1371/journal.pone.0125267.

2. Austrian K, Soler-Hampejsek E, Kangwana B, Wado YD, Abuya B, Maluccio JA. Impacts of two-year multisectoral cash plus programs on young adolescent girls' education, health and economic outcomes: Adolescent Girls Initiative-Kenya (AGI-K) randomized trial. BMC Public Health;21(1):2159. doi:10.1186/s12889-021-12224-3.

3. Bhatia K, Rath S, Pradhan H, Samal S, Copas A, Gagrai S, et al. Effects of community youth teams facilitating participatory adolescent groups, youth leadership activities and livelihood promotion to improve school attendance, dietary diversity and mental health among adolescent girls in rural eastern India (JIAH trial): A cluster-randomised controlled trial. SSM Popul Health;21:101330. doi:10.1016/j.ssmph.2022.101330.

4. Firestone R, Moorsmith R, James S, Urey M, Greifinger R, Lloyd D, et al. Intensive Group Learning and On-Site Services to Improve Sexual and Reproductive Health Among Young Adults in Liberia: A Randomized Evaluation of HealthyActions. Glob Health Sci Pract;4(3):435-51. doi:10.9745/GHSP-D-16-00074.

5. Gourlay A, Birdthistle I, Mthiyane NT, Orindi BO, Muuo S, Kwaro D, et al. Awareness and uptake of layered HIV prevention programming for young women: analysis of population-based surveys in three DREAMS settings in Kenya and South Africa. BMC Public Health;19(1):1417. doi:10.1186/s12889-019-7766-1.

6. Lou CH, Wang B, Shen Y, Gao ES. Effects of a community-based sex education and reproductive health service program on contraceptive use of unmarried youths in Shanghai. J Adolesc Health;34(5):433-40. doi:10.1016/j.jadohealth.2003.07.020.

7. Mehra D, Sarkar A, Sreenath P, Behera J, Mehra S. Effectiveness of a community based intervention to delay early marriage, early pregnancy and improve school retention among adolescents in India. BMC Public Health;18(1):732. doi:10.1186/s12889-018-5586-3.

8. Munea AM, Alene GD, Debelew GT. Quality of youth friendly sexual and reproductive health Services in West Gojjam Zone, north West Ethiopia: with special reference to the application of the Donabedian model. BMC Health Serv Res;20(1):245. doi:10.1186/s12913-020-05113-9.

9. Oberth G, Chinhengo T, Katsande T, Mhonde R, Hanisch D, Kasere P, et al. Effectiveness of the Sista2Sista programme in improving HIV and other sexual and reproductive health outcomes among vulnerable adolescent girls and young women in Zimbabwe. Afr J Aids Res;20(2):158-164. doi:10.2989/16085906.2021.1918733.

10. Patel P, Puwar T, Shah N, Saxena D, Trivedi P, Patel K, et al. Improving Adolescent Health: Learnings from an Interventional Study in Gujarat, India. Indian J Community Med;43(Suppl 1):S12-S17. doi:10.4103/ijcm.IJCM_286_18.

11. Tandon SD, Maulik PK, Tucker MG, Sonenstein F. The mental health needs of out-of-school adolescents and young adults: an intervention conducted in employment training programs, Baltimore, Maryland, 2007-2008. Prev Chronic Dis;9:E69.

12. Riviere C, Bell T, Cadot Y, Perodin C, Charles B, Bertil C, et al. Success of community approach to HPV vaccination in school-based and non-school-based settings in Haiti. PLoS One;16(6):e0252310. doi:10.1371/journal.pone.0252310.

13. Isabirye A, Mbonye M, Asiimwe JB, Kwagala B. Factors associated with HPV vaccination uptake in Uganda: a multi-level analysis. BMC Womens Health;20(1):145. doi:10.1186/s12905-020-01014-5.

14. Msyamboza KP, Mwagomba BM, Valle M, Chiumia H, Phiri T. Implementation of a human papillomavirus vaccination demonstration project in Malawi: successes and challenges. BMC Public Health;17(1):599. doi:10.1186/s12889-017-4526-y.

15. Gallagher KE, Howard N, Kabakama S, Mounier-Jack S, Griffiths UK, Feletto M, et al. Lessons learnt from human papillomavirus (HPV) vaccination in 45 low- and middle-income countries. PLoS One;12(6):e0177773. doi:10.1371/journal.pone.0177773.
